# Ultraviolet B-induced Otx2 expression in lens epithelial cells promotes epithelial–mesenchymal transition

**DOI:** 10.1242/bio.035691

**Published:** 2019-02-04

**Authors:** Yasuo Yoshitomi, Hiromi Osada, Haruka Satake, Masami Kojima, Hidehito Saito-Takatsuji, Takayuki Ikeda, Yoshino Yoshitake, Yasuhito Ishigaki, Eri Kubo, Hiroshi Sasaki, Hideto Yonekura

**Affiliations:** 1 Department of Biochemistry, Kanazawa Medical University School of Medicine, 1-1 Daigaku, Uchinada, Kahoku-gun, Ishikawa 920-0293, Japan; 2 Department of Ophthalmology, Kanazawa Medical University School of Medicine, 1-1 Daigaku, Uchinada, Kahoku-gun, Ishikawa 920-0293, Japan; 3 Department of Ophthalmology, Faculty of Medical Science, Kanazawa Medical University School of Nursing, 1-1 Daigaku, Uchinada, Kahoku-gun, Ishikawa 920-0293, Japan; 4 Medical Research Institute, Kanazawa Medical University, 1-1 Daigaku, Uchinada, Kahoku-gun, Ishikawa 920-0293, Japan

**Keywords:** Cataract, UV irradiation, Otx2, Epithelial–mesenchymal transition (EMT)

## Abstract

Ultraviolet (UV) radiation of eyes is a major risk factor for cataractogenesis, although the molecular mechanisms underlying this process remain poorly understood and genes that are affected by UV radiation have not been fully identified. In this study, we examined the UV-related gene regulation in lens epithelial cells (LECs) of mouse eyes and investigated the molecular mechanisms of UV-triggered cataractogenesis. Forty-one genes were significantly upregulated in LECs following UVB exposure *in vivo* in two independent experiments. Among these, Otx2 was strongly upregulated in LECs, suggesting that it may act as an upstream regulator of UVB-induced changes in gene expression. Accordingly, Otx2 overexpression in LECs *in vitro* induced morphological changes in cell shapes. Epithelial–mesenchymal transition (EMT)-related molecules, such as TGFβ2, αSMA and fibronectin were upregulated in Otx2-overexpressing LECs, concomitant with suppression of lens fiber cell marker genes, such as CRYAA and DNASEIIB. *In vitro* experiments suggested that UVB upregulated Otx2 through hydrogen peroxide generation. Aberrant upregulation of Otx2 in LECs following UV irradiation induces the EMT and alteration of the lens cell characteristics, likely contributing to cataractogenesis.

## Abstract (precis)

**Summary:** UVB irradiation upregulates Otx2 in mouse lens epithelial cells. Otx2 overexpression induces the epithelial–mesenchymal transition and alters gene expression and characteristics of lens cells, likely contributing to cataract formation.

## INTRODUCTION

Cataract is the cause of blindness in 33.4% of cases globally (Khairallah et al., 2015). Previous epidemiological studies show that ultraviolet (UV) exposures of lenses are a major risk factor for cataractogenesis (Bochow et al., 1989). However, the ensuing molecular mechanisms and global transcriptional changes during UV-induced cataractogenesis remain unclear. Hence, studies of the molecular mechanisms of cataractogenesis and the development of noninvasive prevention and treatment methods for cataractogenesis are eagerly awaited.

Eye lenses are uniquely transparent and avascular tissues which are covered with anterior lens epithelia, comprising a single layer of lens epithelial cells (LECs). Lens epithelia are essential for maintaining the homeostasis and optical transparency of the entire lens. However, anterior LECs are continuously exposed to environmental insults such as UV irradiation, which is the major risk factor for cataract. Studies with animal models have shown that UVB irradiation resulted in cataract formation (Andley et al., 1996; Ayala et al., 2000). Several *in vivo* studies have reported gene expression changes in LECs, such as Eaf2 (Jiang et al., 2015) and NKR-1 (Gross et al., 2018), by UVB irradiation and have suggested their involvement in cataract formation. However, the comprehensive gene expression analysis of UVB-exposed lenses has not yet been fully conducted.

Several studies show common phenotypical changes in LECs due to anterior cataracts, including transdifferentiation and fibrosis following the induction of the epithelial–mesenchymal transition (EMT) in LECs (Lee and Joo, 1999; Wang et al., 2017). Anterior subcapsular cataracts are often accompanied by LEC growth and transdifferentiation and consequent production of various non-lens proteins including αSMA (Marcantonio et al., 2003). The EMT is also a characteristic of posterior capsule opacification, which is the most common complication of serious secondary blindness after cataract surgery (Marcantonio et al., 2000; 2003). In addition, transgenic mice expressing transforming growth factor β (TGFβ) under the control of the crystalline promoter were characterized by the EMT in lenses and transdifferentiation of LECs into myofibroblasts (de Iongh et al., 2005). UV radiation reportedly stimulates Snail expression in epidermal keratinocytes and results in induction of the EMT (Hudson et al., 2007). Therefore, UV irradiation likely induces phenotypical changes, such as the EMT in LECs. We previously examined secretory factors that are upregulated by UVB exposures in cultured human LECs, demonstrated UV-induced amphiregulin and GDF15 in LECs, and showed that exogenous amphiregulin significantly activates LEC proliferation (Osada et al., 2011). These UVB-induced protein factors may contribute to pathological changes in lens tissues.

Lens tissue environments are always hypoxic, with oxygen concentrations near lens epithelia of 2% or less (Beebe, 2008). During development, tissues around lens epithelia are enriched in blood vessels, likely maintaining sufficient oxygen concentrations during fetal stages. In contrast, blood vessels retract and lens epithelia are maintained under severe hypoxic conditions after postnatal stages (Ito and Yoshioka, 1999; Zhang et al., 2011). Therefore, further analyses of the effects of UV irradiation on LECs under hypoxic conditions or *in situ* are required.

Herein, we determined for the first time comprehensive gene expression patterns in UV-exposed mouse lens epithelia *in situ* to elucidate the molecular mechanisms of UV-triggered cataractogenesis. A total of 41 genes were identified as UVB responsible in LECs, and Otx2 was shown to be a common upstream transcription factor for these upregulated genes. Accordingly, Otx2 regulates LEC cell morphology, and its overexpression induced EMT-related genes in LECs, resulting in alteration of the lens cell characteristics.

## RESULTS

### Gene expression changes in mouse lens epithelia following UVB irradiation

**Fig. 1.**
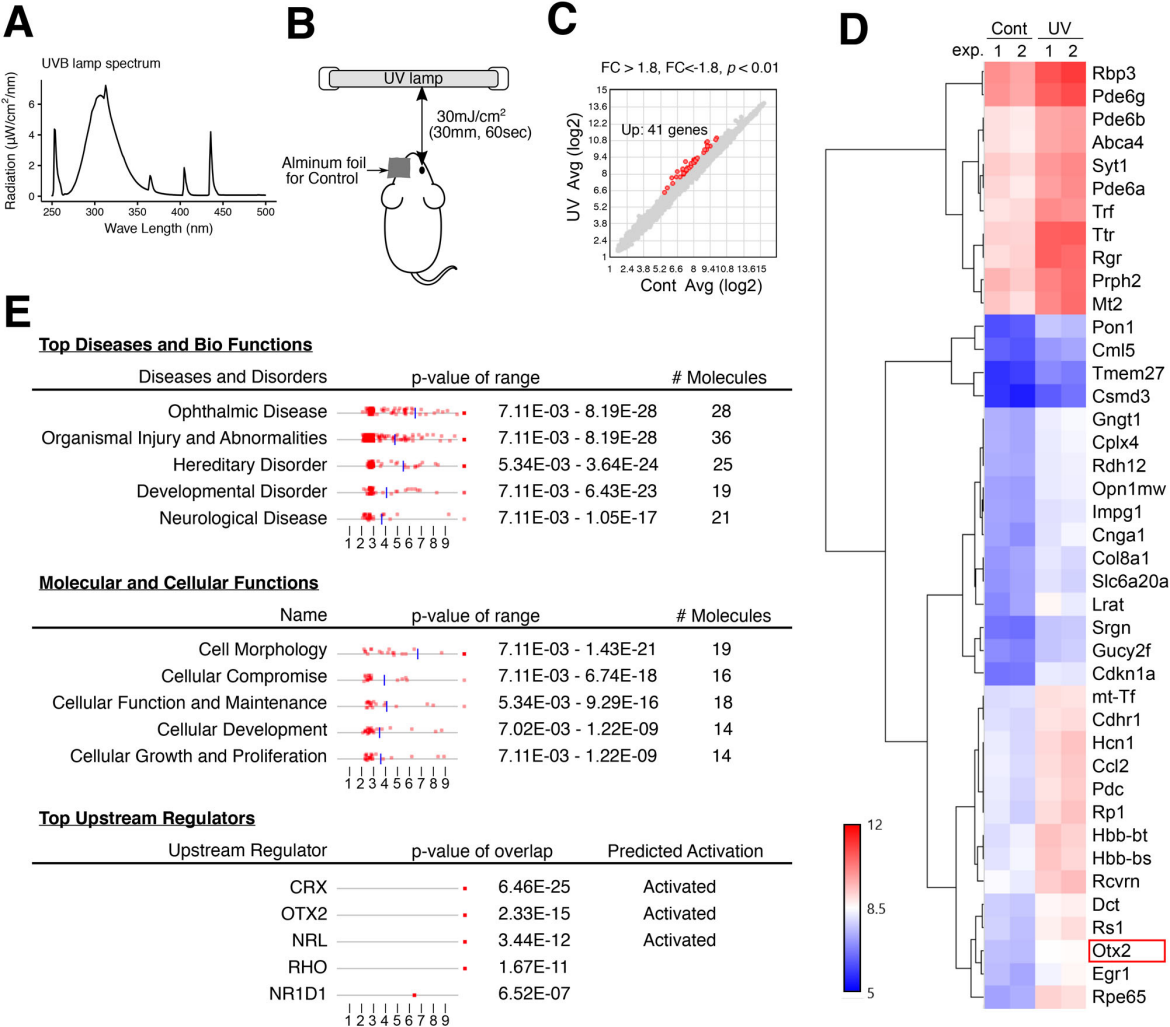
**Gene expression profiling of mouse lens epitheliums with UVB irradiation.** (A) Wave spectrum of the UVB light source used in the radiation experiment were analyzed. (B) Right eyes of mice were exposed to UVB at 30 mJ/cm^2^, while their left eyes were covered with aluminum foil for comparison as controls. (C) Gene expressional profiling of lens epithelium by UVB irradiation in mouse *in vivo* were analyzed by microarray experiments and 41 differentially expressed genes (DEGs) were selected with over 1.8-fold change and *P*<0.01. (D) Normalized signal intensities of DEGs on each gene over two independent experiments are shown in the heatmap. (E) Ingenuity pathway analysis software (IPA) identified the enrichment of DEGs in the specific molecular and functional pathways and the possible upstream regulators among DEGs.

To analyze the effects of UVB irradiation on LECs (Fig. 1), we isolated UVB-irradiated and control lens epithelia from mice and performed microarray analyses (Fig. 1C–E). Genes that met the criteria (>1.8-fold; *P*<0.01. *P* value was used for selection of the commonly up or downregulated genes in the two independent experiments) of differential expression between UV-irradiated and control lens epithelia from two independent experiments included 41 that were upregulated by UVB (Fig. 1C,D), although no downregulated genes were observed under these conditions (Fig. 1C). Subsequent gene ontology analyses of UVB-induced genes using ingenuity pathway analysis (IPA) software indicated significant representation of ophthalmic disease-related and cell morphology-related genes (7.11 E-03>P>8.19 E-28 and 7.11 E-03>P>1.43 E-21, respectively). Moreover, the IPA software indicated that the homeobox protein Otx2 is an upstream regulatory transcription factor that is sensitive to UVB irradiation (*P* value of overlap=2.33 E-15; Predicted Activation State, Activated; Fig. 1D). The cone-rod homeobox protein Crx was also predicted as an upstream transcription factor but was positioned downstream of Otx2 in previous signaling pathway analyses (Omori et al., 2011). Otx2 is a member of the bicoid sub-family of homeodomain-containing transcription factors and is expressed in developing brain tissues as a key regulatory gene during the acquisition of visual plasticity (Sugiyama et al., 2008). Wyatt et al. analyzed the OTX2 gene in 165 patients and correlated various mutations with ocular malformations, including anophthalmia, microphthalmia and coloboma (Wyatt et al., 2008). However no available reports describe Otx2 functions in lens epithelia.

### UVB irradiation induces Otx2 expression in LECs

**Fig. 2.**
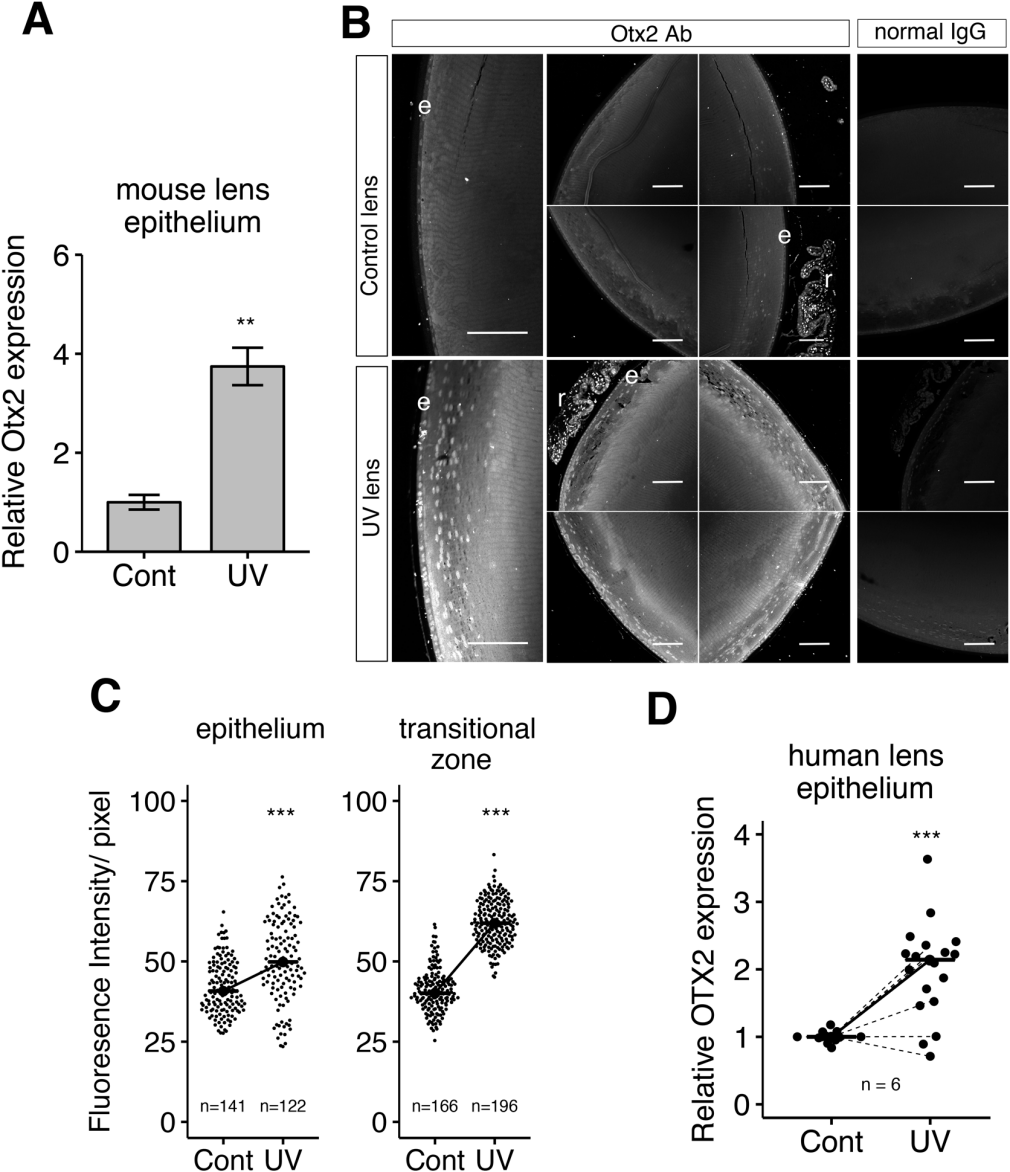
**Otx2 expression in mouse and human lens epitheliums with or without UVB irradiation.** (A) Otx2 expression in lens epithelial cells (LECs) with (UV) or without UVB irradiation (Cont) was confirmed by RT-qPCR. (B) Mouse lens sagittal sections from *in vivo* UVB irradiation experiments containing LECs of the surface and transitional zones were stained with anti-Otx2 antibody or normal IgG as a negative control, and fluorescent images were captured by LSM 710 confocal microscopy. e, LECs; r, retinal cells. Scale bars: 100 µm. (C) Individual fluorescence intensities of cells in Otx2 staining of five randomly selected images were quantified and their lens epithelia (surface area) and transitional zones were compared. The number of quantified cells is indicated. The similar results were obtained in three independent experiments. (D) Human lens epitheliums from six patients were collected and subjected to the UVB irradiation experiment. Otx2 expression was analyzed by RT-qPCR and quantified. Average of three measurements was recorded for each half. For the plots in C and D, all data points are indicated as dots and medians are indicated as horizontal bars connected by a solid diagonal line between the samples. Dashed lines in D indicate expressional differences in the median of each patient pair measured by RT-qPCR. **P<0.01, ***P<0.001.

Initially, we confirmed that Otx2 mRNA expression is induced by UVB irradiation in mouse LECs using RT-qPCR (Fig. 2A) and subsequently investigated Otx2 protein expression in mouse LECs using immunohistochemical methods. These analyses of mouse lens tissue sections revealed that Otx2 is expressed in retinal cells and in LECs (Fig. 2B), as described previously (Nishida et al., 2003). Otx2 expression levels were much higher in UVB-irradiated LECs than non-irradiated control LECs (Fig. 2B). Otx2 upregulation in UVB-irradiated lens was observed in lens surface areas and also in transition zones which contained proliferating and differentiating LECs (Fig. 2B,C). These observations agree with the present microarray analyses of UVB-irradiated LECs (Fig. 1D) and suggest that UVB-induced Otx2 expression alters the LEC phenotype. No morphological differences were noted in the cell shapes or epithelium thickness between the UVB-irradiated lenses and control lenses at 24 h of UVB irradiation.

In further studies, we determined whether UVB irradiation induces Otx2 expression in human lens epithelia using human anterior lens capsular epithelial tissues that were isolated from patients during cataract surgery. Patient and sample information is described in Table S2. After exposing these human lens epithelial tissues to UVB *ex vivo*, significant Otx2 induction in human LECs was confirmed in four of six individuals by RT-qPCR, as described in the Materials and Methods section (Fig. 2D).

**Fig. 3.**
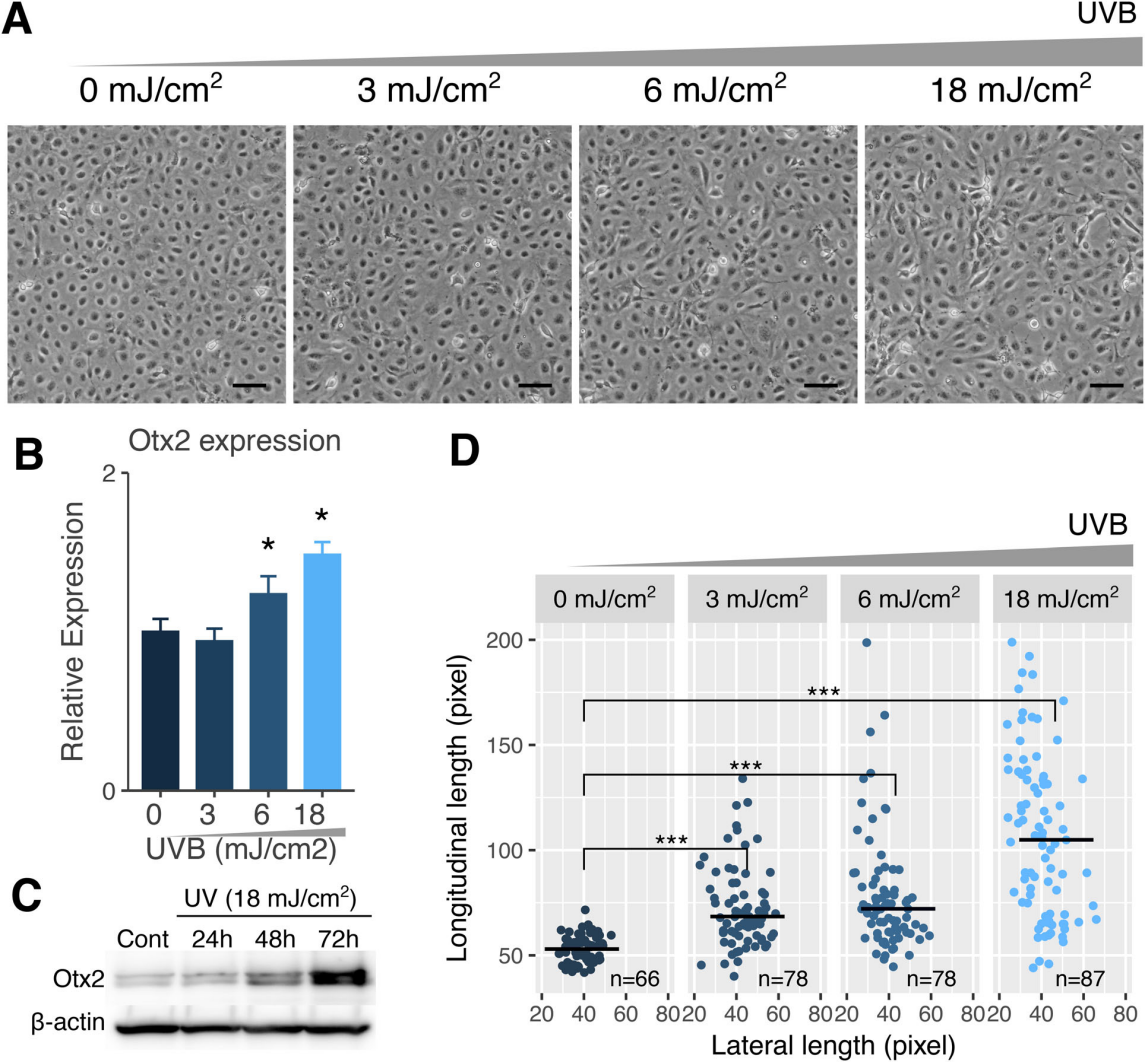
**UVB irradiation induces Otx2 expression and changes in cell morphology of human lens epithelial SRA cells.** (A) Representative phase-contrast images of SRA cells exposed to UVB at doses of 0, 3, 6 and 18 mJ/cm^2^. Scale bars: 100 µm. (B) Otx2 expression in UVB-irradiated SRA cells was quantified by RT-qPCR. (C) Otx2 protein expression in UVB-irradiated SRA cells at 18 mJ/cm^2^ was analyzed by western blot. (D) Longitudinal and lateral lengths of cells were measured and plotted. *P<0.05, ***P<0.001.

To verify dose dependency of UVB irradiation on Otx2 induction in LECs, we next analyzed Otx2 expression under different UVB irradiation exposure using immortalized human lens epithelial cell line SRA 01/04 cells (SRA cells) (Fig. 3A). At 24 h of irradiation with 0, 3, 6 and 18 mJ/cm^2^ UVB, Otx2 expression was upregulated in a UVB dose-dependent manner (Fig. 3B). We also confirmed Otx2 induction at protein levels by western blot analysis in UVB-irradiated cells (Fig. 3C, ∼3.9-fold versus control cells). These induction levels were comparable against the induction levels *in vivo* mouse lenses by UVB-irradiation. We noticed that SRA cells were elongated, similar to fibroblast cells, after UVB irradiation and that this elongation occurred in a UVB dose-dependent manner (Fig. 3D).

### Overexpression of Otx2 in SRA cells leads to the development of fibroblast morphologies

**Fig. 4.**
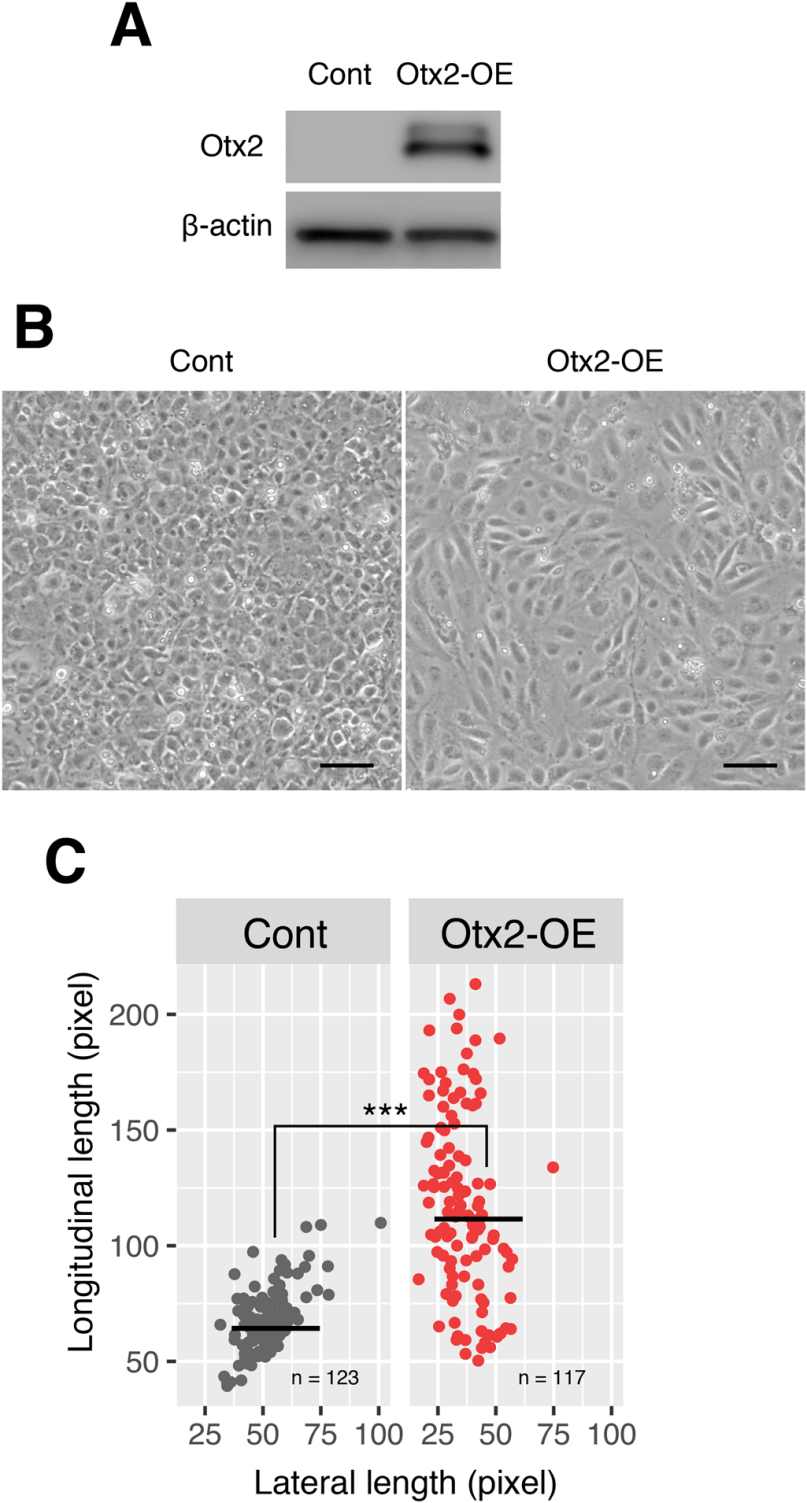
**Overexpression of Otx2 in SRA cells changes their cell morphology.** (A) Otx2 expression of Otx2-overexpressing SRA cells (Otx2-OE) and control cells (Cont) were analyzed by western blot with anti-Otx2 antibody (54-fold versus control cells). β-actin expressions in each sample were used for loading controls in western blot. (B) Representative phase-contrast images of Otx2-overexpressing SRA cells (Otx2-OE) and control cells (Cont) are shown. The same results were obtained in five independent experiments. Scale bars: 100 µm. (C) Longitudinal and lateral lengths of cells were measured and plotted. ***P<0.001.

To investigate the effects of Otx2 overexpression in LECs, we performed experiments with SRA cells. Initially, we confirmed lentivirus-mediated overexpression of Otx2 (Otx2-OE) in SRA cells using western blotting (Fig. 4A). Furthermore, transduction efficiency was confirmed by GFP expression, which was produced from the lentivirus vector backbone (Fig. S2A). There was no difference in cell numbers between Otx2-OE cells and control cells (Fig. S2C). Otx2-OE cells induced morphological changes after 2 days of transduction (Fig. 4B). Similar to UVB-irradiated-SRA cells depicted in Fig. 3A, the cell shapes were elongated and resembled fibroblasts, with significantly increased lengths of the longitudinal axes of Otx2-OE cells as compared with the polygonal shapes of control epithelial cells (Fig. 4C). These results suggest that Otx2 overexpression induces the EMT in LECs.

### Otx2 overexpression induces the EMT in LECs

**Fig. 5.**
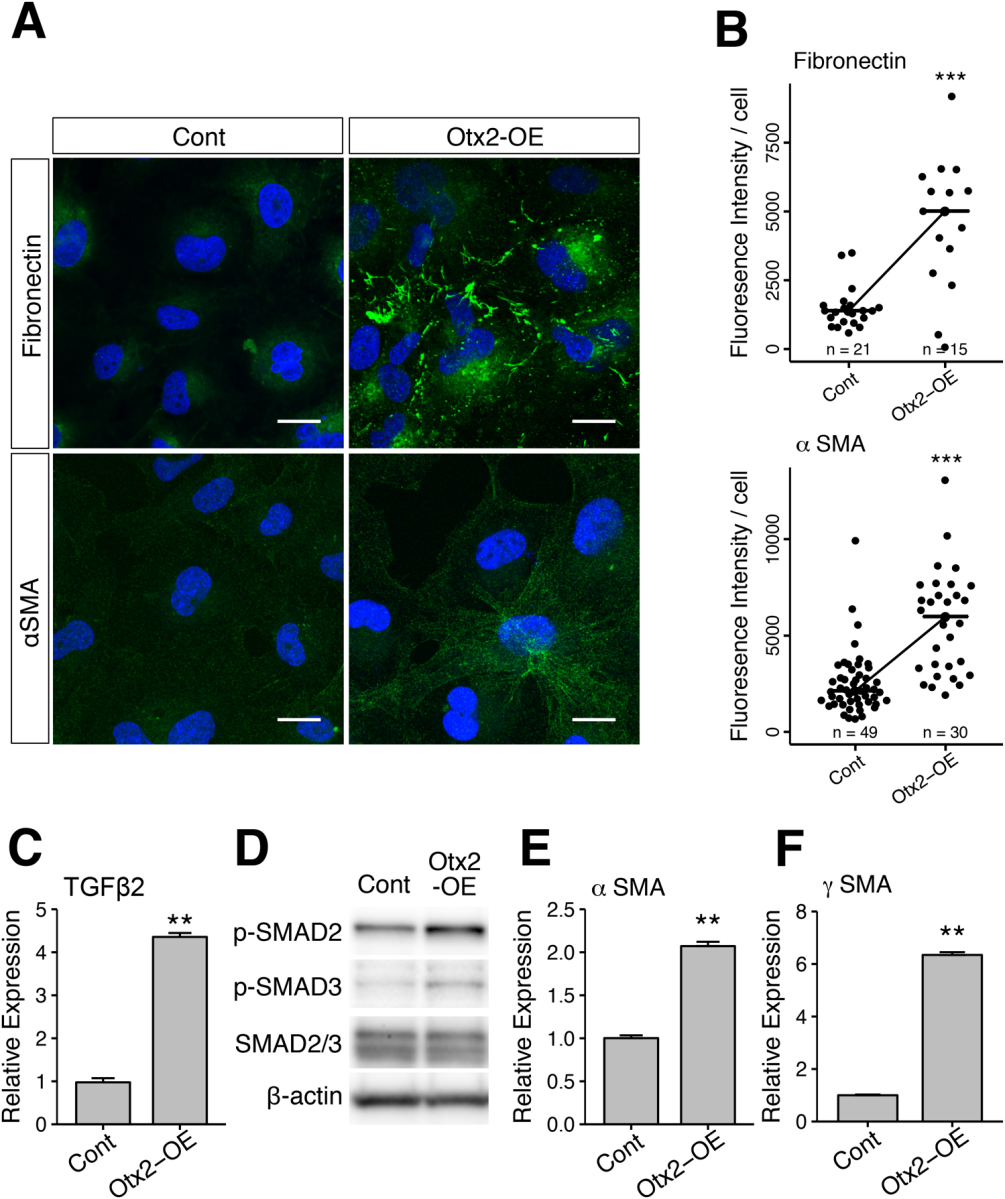
**Epithelial-mesenchymal transition (EMT) in Otx2-OE lens epithelial cells.** (A) Otx2-overexpressing cells (Otx2-OE) and control cells (Cont) were stained with anti-fibronectin or anti-α-SMA antibodies (green) and with the nuclear stain DAPI (blue). Scale bars: 20 µm. (B) The fluorescent intensities of anti-fibronectin and anti-α-SMA signals per cell area in five randomly selected images were measured and quantified. Similar results were obtained in three independent experiments. Data points of measurement are indicated as dots and medians are indicated by horizontal bars. (C) TGFβ2 mRNA expression in Otx2-OE cells was analyzed by RT-qPCR. (D) Phosphorylation of Smad 2 (p-SMAD2) and 3 (p-SMAD3) was analyzed by western blot. Smad2/3 and β-actin expression in each sample were used for loading controls in western blotting. (E,F) mRNA expression of α-SMA (E) and γ-SMA (F) in Otx2-OE cells were analyzed by RT-qPCR. **P<0.01, ***P<0.001.

Four days after Otx2-lentivirus-mediated transduction in LECs, we stained the mesenchymal differentiation markers fibronectin and αSMA with respective antibodies (Fig. 5A,B) and showed that Otx2-OE cells express these proteins abundantly, whereas no staining was observed in control cells (Fig. 5A). Further quantitative analyses of fluorescence signals confirmed that fibronectin and αSMA expression levels were significantly higher in Otx2-overexpressing cells than in control cells (Fig. 5B). To investigate the involvement of TGFβ-signaling cascade in Otx2-induced EMT in LECs, we examined TGFβ2 expression and detected TGFβ2 induction in Otx2-OE cells by RT-qPCR (Fig. 5E,F). As shown in Fig. 5D, phosphorylated Smad 2 and 3, which are the TGFβ2 pathway components, were increased in Otx2-OE SRA cells, suggesting that TGFβ-signaling cascade was activated by Otx2. In addition, RT-qPCR analyses showed corresponding increases in αSMA and γSMA mRNA expression levels (Fig. 5C), further indicating that UV-mediated upregulation of Otx2 induces the EMT and abnormal mesenchymal differentiation of LECs. In the microarray analysis using RNA at 24 h after UVB exposure, fibronectin, αSMA and γSMA showed an increasing trend albeit not over 1.8 logFC difference as compared to the control. It may thus take more time to reach the full activation of these EMT markers.

### Otx2 suppresses the expression of lens markers of differentiation in SRA cells

**Fig. 6.**
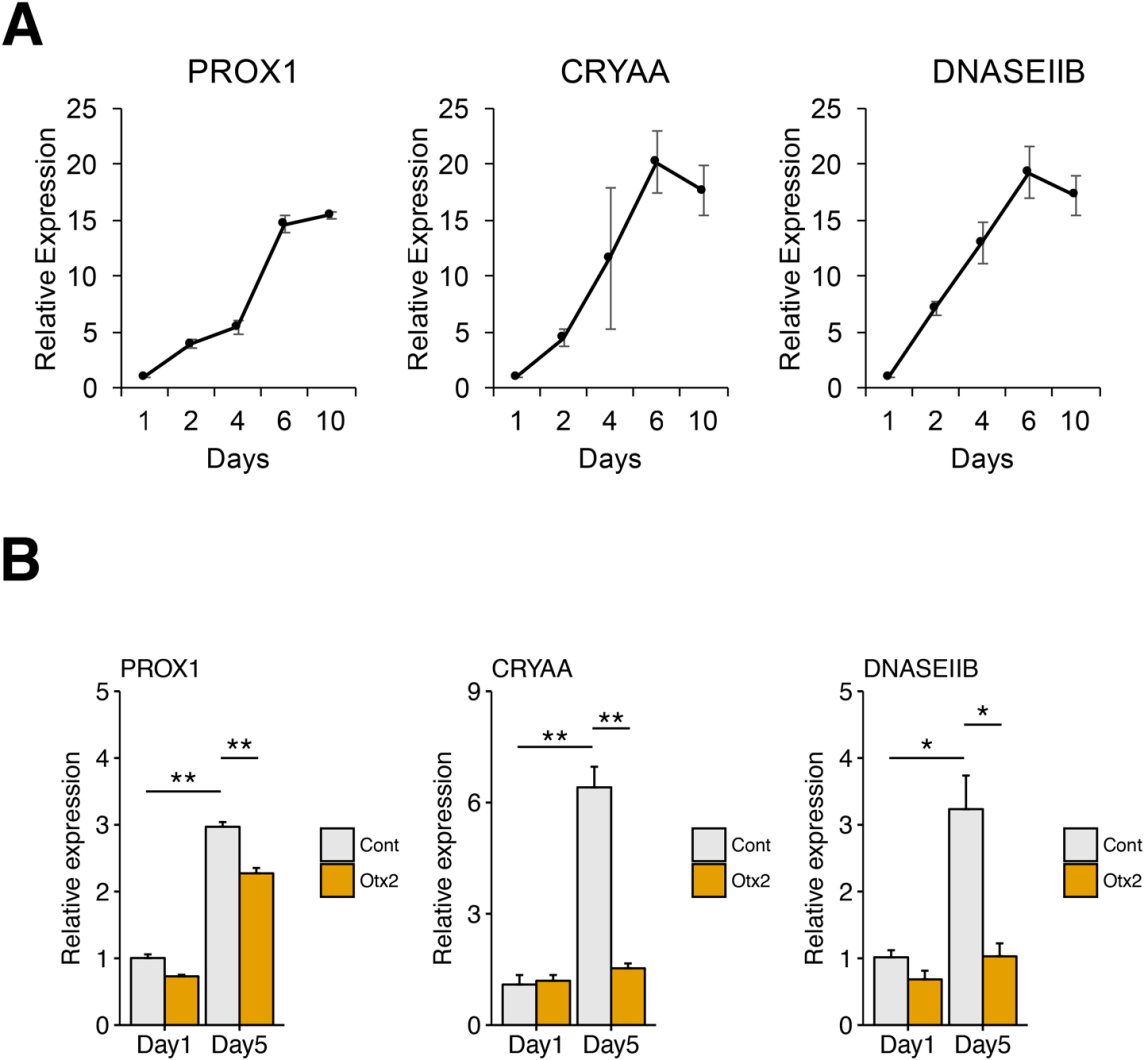
**Otx2 changes gene expression in SRA cells in the lens fiber differentiation culture.** (A) SRA cells were cultured in the differentiation conditions described in the Materials and Methods section for 10 days and mRNA expression of PROX1, CRYAA and DNASEIIB on day 1, 2, 4, 6 and 10 were measured by RT-qPCR. (B) Otx2-overexpressing cells (Otx2-OE) and control cells (Cont) were cultured in the same differentiation conditions in A and at day 1 and day 5 mRNA expression of PROX1, CRYAA and DNASEIIB were analyzed by RT-qPCR. *P<0.05, **P<0.01.

To assess the effect of aberrant Otx2 upregulation by UV-exposure in lens cells, we analyzed gene expression changes of lens cells with Otx2 upregulation. LECs reportedly differentiate into lens fiber cells in the presence of FGF2 and BMP4 (Belecky-Adams et al., 2002). We then induced gens characteristic to lens fiber cells in SRA cells in the presence of FGF2 and BMP4 for 10 days and analyzed gene expressions. Under these conditions, increased expression of gens characteristic to lens fiber cells was observed over 10 days, including the differentiation initiation marker Prox1 (Lang, 2004) and the terminal differentiation markers Crystalline αA (CRYAA; Chow and Lang, 2001) and DNase IIB (DNASE2B; De Maria and Bassnett, 2007; Fig. 6A). To elucidate the effect of Otx2 in lens fiber cells, we assessed these gene expression levels in Otx2-overexpressing cells on day 5 (Fig. 6B). The ensuing data suggested that ectopic Otx2 induction suppresses the gene expression of CRYAA and DNASEIIB. Prox1 expression was significantly lower than that in control cells, but it was still activated in Otx2-OE cells. The results indicated that Otx2 affects the expression of some lens fiber marker genes such as CRYAA and DNASEIIB.

### Otx2 induction by UVB irradiation in SRA cells is mediated by H_2_O_2_

**Fig. 7.**
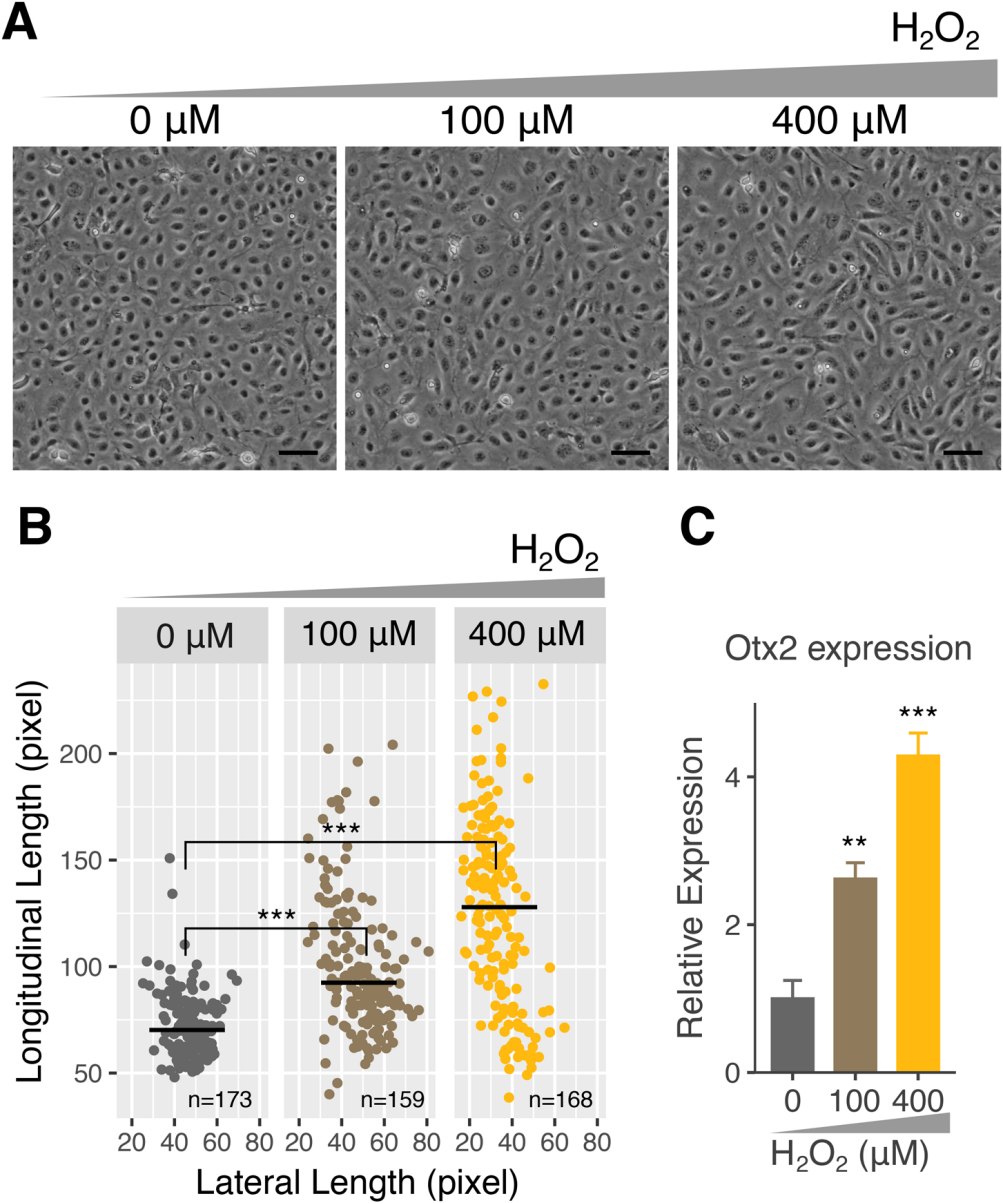
**Effects of H_2_O_2_ treatment on Otx2 expression and cell shapes in SRA cells.** (A) SRA cells were treated with 0, 100 and 400 µM of H_2_O_2_ in PBS for 30 min. Representative phase-contrast images of H_2_O_2_-treated cells were captured at 48 h post treatment. Scale bars: 100 µm. (B) Longitudinal and lateral lengths of each cell were measured and plotted. (C) Otx2 expression of H_2_O_2_-treated cells was quantified by RT-qPCR. **P<0.01, ***P<0.001.

UVB irradiation on eye lens results in the generation of H_2_O_2_, which is secreted into the aqueous humor (Ivanov et al., 2018). To investigate the induction mechanisms of Otx2 by UVB irradiation, we examined effects of H_2_O_2_ on Otx2 expression and cell morphology in SRA cells. As shown in Fig. 7A, H_2_O_2_ treatment induced morphological changes in SRA cells, as observed in UVB-irradiated (Fig. 3) and Otx2-OE cells (Fig. 4). The cells were elongated by H_2_O_2_ treatment in a dose-dependent manner (Fig. 7B). Moreover, H_2_O_2_ treatment upregulated the Otx2 expression in SRA cells in a dose-dependent manner (Fig. 7C). These results suggest that UVB-induced Otx2 expression is mediated by H_2_O_2_, which was generated by UVB irradiation.

## DISCUSSION

Although UV irradiation is the major risk factor for cataractogenesis, the related molecular mechanisms remain poorly understood. Herein, we show that UVB irradiation of eye lenses upregulates Otx2 in LECs and that Otx2 induced EMT in lens epithelial cells, resulting in alteration of lens cell characteristics.

Initially, we performed microarray experiments to identify genes that are influenced by UV irradiation in eyes *in vivo* and found 41 DEGs with potential roles in UVB-directed cataractogenesis (Fig. 1). All 41 genes were upregulated, and no genes were downregulated by UVB irradiation in LECs, suggesting that UVB irradiation predominantly activated genes in eye lens. Subsequent gene ontology analyses using IPA software identified Otx2 as a dominating upstream regulator of these 41 candidate genes (Fig. 1E). Otx2 is as an upstream regulator of the transcription factor CRX. Thus, in subsequent experiments, we examined functions of Otx2 in LEC cell response to UVB exposure. Accordingly, Otx2 was previously related to vertebrate brain and retinal development, and phenotypes that have been associated with heterozygous Otx2 mutations and whole deletion mutations include anophthalmia, microphthalmia and retinal dystrophy (Ragge et al., 2005; Wyatt et al., 2008). Although Otx2 is expressed in differentiated retinal photoreceptors (Fossat et al., 2007), the functions of Otx2 in lens cells have not been examined previously. We found that several of the upregulated genes were related to the retina (e.g. Opn1mw, Pde6b, Pde6g and Gngt1). We believe that the upregulation of these retina-related genes was due to Otx2 overexpression, since we focused upon the isolation of the lens epithelia to avoid the contamination of other retinal tissues. Moreover, the upregulated genes in Otx2-overexpressing SRA cells considerably overlapped with the genes upregulated in UVB-irradiated lens epithelium (data not shown), which is consistent with the report that Otx2 is involved in retinal development. Interestingly, another transcription factor, Egr1, was also upregulated by UVB irradiation (Fig. 1D). Egr1 is an important regulator for LEC cell viability and fibrosis, whose expression is regulated by integrin beta-1 (Wang et al., 2017). The examination of effects of Egr1 overexpression in LECs is warranted to elucidate molecular mechanisms underlying cataract formation.

The present immunohistochemical analyses of Otx2 in lens epithelia indicate that Otx2 is expressed at low levels in LECs but is strongly induced by UVB in both the surface and equator transitional zones (Fig. 2). We confirmed these data from UV-exposed mouse lenses in human LECs of anterior capsules from cataract surgery patients and again observed strong induction of Otx2 following exposures to UVB irradiation (Fig. 2D). Using the human lens epithelial cell line SRA, we found that Otx2 was induced at both mRNA and protein levels in a UVB dose-dependent manner (Fig. 3B). In addition, morphological changes in UVB-irradiated cells to fibroblast-like cells occurred in a UVB dose-dependent manner (Fig 3A–C). The results suggest that Otx2 could induce EMT even at the lower levels.

To investigate Otx2 function in LECs, we transduced Otx2 in SRA cells. As shown in Fig. 4, Otx2-overexpressing LECs exhibited changes from epithelial polygonal shapes to fibroblast morphologies as in the UVB-irradiated cells (Fig. 3A), and the mesenchymal markers fibronectin and αSMA were induced in LECs at 4 days after transduction of Otx2-expressing vectors (Fig. 5A). Collectively, these data suggest that UVB irradiation induces Otx2, which then mediates EMT-related changes in LECs. Thus, to determine whether Otx2-mediated EMT of LECs affects the expression of lens fiber cell marker genes, we examined the expression of CRYAA and DNase IIB in control and Otx2-OE cells. We observed the induction of these genes in control cultures on days 5–10 (Fig. 6A). In Otx2-OE cells, CRYAA and DNASEIIB were largely suppressed (Fig. 6B). Taken with the relative absence of these proteins in Otx2-overexpressing LECs (Fig. 6B), these data indicate that Otx2 changes lens cell characteristics. Prox1 has roles for FGF-induced lens fiber differentiation of LECs (Audette et al., 2016). Prox1 expression in Otx2-OE cells was significantly lower than control cells at day 5, but it was still activated, suggesting that Otx2 does not totally inhibit lens fiber cell differentiation, but may increase the lens cells of altered characters.

The data presented herein suggest that Otx2 induces the EMT in LECs, resulting in accumulation of lens cells of altered characteristics. Because TGFβ reportedly induced the EMT in lens tissues (de Iongh et al., 2005), we determined TGFβ expression levels in the present Otx2-OE cells. In accordance with our hypothesis that Oxt2 induces the EMT, TGFβ2 expression was 4.5-fold greater in Otx2-OE cells than in control cells (Fig. 5C) and downstream-signaling molecules phosphorylated Smad 2 and 3 were increased by Otx2 overexpression (Fig. 5D), suggesting that UV-induced Otx2 promotes the EMT by upregulating TGFβ. However, TGFβ was not induced significantly in mouse LECs at 24 h after UV irradiation (Fig. 1), potentially reflecting more rapid and transient peak TGFβ expression, as indicated by clear induction of TGFβ3 in human LECs at 12 h after UV exposures and subsequent reversion at 24 h post UV irradiation (Osada et al., 2011). Collectively, the present and previous data suggest that Otx2-dependent TGFβ upregulation contributes to the EMT in LECs. Accordingly, among 11 genes that were upregulated in UV-treated and Oxt2-overexpressing cells, the EMT-related genes Ccl2 (Zhuang et al., 2018), Cldn1 (Zhao et al., 2015) and BMP2 (McCormack and O'Dea, 2013) were identified (data not shown). Vitronectin, which reportedly induces EMT in LECs in the lens epithelial explant (Taliana et al., 2006), was also upregulated in UV-treated and Otx2-overexoressing cells (data not shown). Correlations of EMT with cataractogenesis have been reported previously (de Iongh et al., 2005), further associating these molecules with EMT induction and suggesting that Otx2-mediated induction of the EMT is a predominating process during UV-related cataractogenesis.

In this study, we observed strong induction of Otx2 in the surface area and, notably, also in the equatorial transitional zone of the UVB-irradiated lens. Similarly, Ayala et al. (2000) have reported that UVB exposure to eye lenses *in vivo* induces apoptosis in both the central and equatorial lens epithelia. These effects possibly occur due to the indirect effects from the UVB-exposed cells, such as UV-light-induced oxidative stress, because LECs in the equatorial transitional zone are supposed to be protected against direct UVB exposure by the iris in the eyes. Reportedly, UVB radiation is an efficient generator of H_2_O_2_ (Ivanov et al., 2018). In addition, recent studies have demonstrated that relatively long half-life and non-radical H_2_O_2_ act as a second messenger of redox signaling (Rhee, 2006) and that H_2_O_2_ can be secreted and transported between cells via the plasma membrane aquaporins (Bienert and Chaumont, 2014). H_2_O_2_ also regulates the expression and activity of transcription factors (Suzuki and Yamamoto, 2017). Therefore, we finally analyzed the involvement of H_2_O_2_ in UVB irradiation-triggered Otx2 upregulation and EMT induction. H_2_O_2_ treatment induced morphological changes in a dose-dependent manner, which was concomitant with Otx2 induction in LECs (Fig. 7), which are similar to effects noted in UVB-irradiated LECs (Fig. 3). Based on these results, we hypothesize that UVB irradiation produces H_2_O_2_ in the anterior surface and that H_2_O_2_ is secreted and transported into the equatorial transitional zone and induces the Otx2 overexpression, following which Otx2 induces EMT and alters lens fiber phenotypes, thereby leading to the opacity of the lens and cataract formation. This study also suggests the importance of the signal transduction from central lens epithelial cells to the equatorial lens cells by UVB-induced hydrogen peroxide in cataract formation.

In summary, we demonstrated that UVB exposure upregulates Otx2 expression in LECs and that Otx2 induces the EMT and alters lens cell characteristics. Because these Otx2-related mechanisms likely contribute to UV-triggered cataractogenesis, the present data clarify the pathogenesis of UV-related cataracts and other lens-related diseases and could be used to develop preventive treatments for cataract.

## MATERIALS AND METHODS

### UV irradiation of mouse eye lenses *in vivo*, and human LECs in *ex vivo* cultures

All animal procedures were performed in accordance with the Kanazawa Medical University Animal Guidelines under the authority of project license no. 2016-43. All animal experiments were performed in duplicates, using 8-week-old mice (*n*=3). *In vivo* UVB irradiation of mouse eyes was performed using a 15 W UVB FL-15E bulb (Tozai Elec. Indus., Osaka, Japan). Wave spectra of UVB lamp sources were initially determined using a photospectrometer, and major peak emissions at 313 nm were confirmed (Fig. 1A). Anesthesia was achieved using mixtures containing 0.3 mg/kg medetomidine, 4.0 mg/kg midazolam and 5.0 mg/kg butorphanol, and right eyes of 8-week-old C57BL/6 male mice were then exposed to UVB at 30 mJ/cm^2^ for 60 s at 30 mm from the light source. Left eyes of the same mice were covered with aluminum foil for use as controls. The radiation energy of these conditions was previously evaluated using a UVX Radiometer (UVP, Upland, CA) with a UVX-31 Sensor (UVP), and distances and times were adjusted accordingly. UVB-irradiated lenses and control aluminum foil-covered lenses of the same mice were comparatively analyzed. Twenty-four hours post UVB irradiation, mice were euthanized, eyes were removed immediately, and lenses were dissected using a posterior approach followed by careful and complete removal of the other tissues to avoid contamination (e.g. retinal tissues). Cortex and nucleus portions were also removed and lens epithelia were isolated. Total RNA was then extracted from the lens epithelia for use in microarray and RT-qPCR analyses.

Human lens epithelia were obtained from routine uneventful cataract surgery at the Kanazawa Medical University Hospital (age: 47–86 years, *n*=6) under licensed protocols and were then stored in Dulbecco's minimum essential medium (DMEM) supplemented with 10% fetal bovine serum (FBS) and penicillin/streptomycin until use in experiments. After brief washing in ice-cold HBSS, anterior subcapsular epithelium tissues of each patient were divided into two pieces using a stereo microscope and were separated into control and UVB irradiation groups. UVB exposures of human lens epithelia in ice-cold HBSS were performed at 15 mJ/cm^2^ for 30 s at 30 mm from a light source with a 295 nm sharp band-cut filter (WG295, 100 mm square; Shibuya Optical, Wako, Japan), and samples were then cultured in 10% DMEM in an incubator containing 5% CO_2_. Control pieces were similarly cultured but without UVB irradiation. Under natural UVB exposure conditions, about 60% of the UVB light is absorbed by the cornea and other tissues prior to reaching lens epithelia. Therefore, *in vitro* UVB irradiation experiments with human lens epithelia were performed at 15 mJ/cm^2^ with a 295 nm band-cut filter, resulting in about half of the energy applied in *in vivo* UVB irradiation experiments (30 mJ/cm^2^), but corresponding with UVB energy equivalents on LECs *in vivo*. UVB light wave spectra with the 295 nm band filter were previously determined using a spectroradiometer (USR-40D, Ushio, Japan; see Fig. S1). After 24 h culture, total RNAs were isolated for microarray and RT-qPCR analyses. Human lens epithelia were collected from six individuals, and Otx2 expression was analyzed with or without UVB irradiation. Studies using human lens epithelia were performed with approval from the Kanazawa Medical University Research Ethics Committee (License No. G134). Informed consent was obtained from each participant prior to the study. All procedures conformed to the tenets of the Declaration of Helsinki.

### Microarray analysis

Microarray analyses were performed as described previously (Yoshitomi et al., 2017). Briefly, total RNA was extracted using TRIZOL reagent (Invitrogen) and was purified using RNeasy Mini kits (Qiagen, Valencia, CA). Subsequently, 100 ng aliquots of total RNA were labeled using GeneChiP WT Sense Target Labeling kits according to the manufacturer's instructions (Affymetrix, Santa Clara, CA). Fragmented and labeled cDNAs were then hybridized onto Affymetrix GeneChip Human Gene 1.0 ST arrays, which were then washed and stained using a GeneChip Fluidics Station 450 and scanned using a 3000 7G GeneChip Scanner (Affymetrix). All arrays passed the quality control criteria of the Affymetrix Expression Console software. After normalizing CHP files, differentially expressed genes (DEGs) with >1.8-fold changes and *P*-values <0.01 in two independent experiments were selected using a Transcriptome Analysis Console (Applied Biosystems, Thermo Fisher Scientific, Rockford, IL).

### Cell culture and UV irradiation

SRA01/04 immortalized human LECs (SRA cells) were obtained from Dr. Nobuhiro Ibaraki (Department of Ophthalmology, Jichi Medical University, Tochigi, Japan) and were maintained in DMEM containing 20% FBS and penicillin/streptomycin in an incubator containing 5% CO_2_.

In the experiments of lens fiber cell marker gene induction, SRA cells were seeded in 24-well plates at semi-confluent densities and were cultured overnight. Culture media were then replaced with differentiation medium containing 35 ng/mL BMP4 (ReproTech, MN), 250 ng/mL FGF2 (Sigma-Aldrich, and 0.5 µg/mL heparin (Sigma-Aldrich) in SRA medium.

SRA cells in ice-cold PBS were exposed to 0, 3, 6 and 18 mJ/cm^2^ UVB using a 295-nm band-cut filter. After 24 h of culture in the growth medium, phase-contrast images were captured and total RNA was isolated after 72 h of culture for RT-qPCR analyses.

### H_2_O_2_ treatment of SRA cells

SRA cells in 6-well plate were treated with 0, 100 and 400 µM of H_2_O_2_ in PBS for 30 min in a CO_2_ incubator. After being briefly washed with PBS, cells were cultured for 24 h in the growth medium. After 48 h of H_2_O_2_ treatment, phase-contrast images were captured for cell morphological analysis. RNA was purified from control and H_2_O_2_-treated SRA cells and subjected to RT-qPCR analysis.

### Plasmid vectors and lentivirus production

Lentiviral vector production was performed as described previously (Yoshitomi et al., 2017). Briefly, the human Otx2 (RefSeq NM_001286481.1) open reading frame was amplified using RT-PCR using primers listed in Table S1 and was cloned into pCDH vectors (System Biosciences, Palo Alto, CA). Sequences were then verified using a 3500XL sequencer (ABI), and lentivirus particles were propagated in HEK293TN cells (System Biosciences) following transfection with the pMD2.G envelope vector and the packaging psPAX2 vector (deposited by Didier Trono, Laboratory of Virology and Genetics, Ecole Polytechnique Federale de Lausanne, Switzerland; Addgene plasmid #12259 and #12260, respectively, Addgene, MA). Supernatants from 48 h cultures were collected, and then lentiviral particles were concentrated using PEG-it solution (System Biosciences). Lentivirus infectious units (IFU) were then determined according to numbers of GFP-positive SRA cells, as assessed using serial dilutions of lentivirus from cells at 24 h post transduction.

### RT-qPCR

Total RNA was extracted from cells as described above, and 1 µg aliquots were reverse transcribed using PrimeScript RT Reagent Kit (Takara, Otsu, Japan). The resulting cDNAs were then analyzed using qPCR with an initial denaturation step at 95°C for 30 s, followed by 45 cycles of 95°C for 5 s and 60°C for 30 s using TB Green Fast qPCR Mix (TaKaRa) with the primers listed in supplemental Table 1 and a StepOnePlus Real-Time-PCR System (Applied Biosystems, CA).

### Western blotting

Western blotting was performed as described previously (Yoshitomi et al., 2017). Briefly, cells were lysed in Laemmli buffer containing 5% 2-mercaptoethanol and were sonicated for 30 s and then heated at 98°C for 5 min. The resulting protein samples were subjected to SDS-PAGE using 4%–12% polyacrylamide gels (Wako, Osaka, Japan). After electrophoresis, proteins were transferred to polyvinylidene difluoride membranes (Invitrogen) and were blocked with 2% BSA in Tris-buffered saline. Membranes were then incubated with Anti-Otx2 antibody (cat. no. AF1979; R&D Systems, Minneapolis, MN), anti-Phospho-Smad2 (Ser465/467) (Clone no. 138D4, Cat. no. 3108, 1:1000, Cell Signaling Technology, Danvers, MA), anti-Phospho-Smad3 (Ser423/425) (Clone no. C25A9, Cat. no. 9520, 1:1000, Cell Signaling Technology), anti-Smad2/3 (Clone no. D7G7, Cat. no. 8685, 1:1000, Cell Signaling Technology), and anti-β-actin (Cat. no. A2228, 1:4000; Sigma-Aldrich) primary antibodies, followed by appropriate HRP conjugated secondary antibodies (Amasham GE, MA). Protein signals were then detected using ECL Plus Chemiluminescent Substrate (Thermo Fisher Scientific, Rockford, IL) with a Vilber-Lourmat FUSION FX7 imaging system (Vilber, Collégien, France).

### Immunohistochemistry and immunocytochemistry

Mouse lens tissues were fixed with 4% paraformaldehyde in phosphate-buffered saline (PBS) overnight, washed three times in PBS, and were then stored in 70% ethanol. Paraffin-embedded lens sections were then rehydrated and blocked with 10% goat serum in PBS containing 0.01% Triton X-100 (PBST). Tissue sections were then incubated overnight with anti-Otx2 antibody (AF1979, 1:100; R&D Systems) at 4°C. Slides were washed with PBS and incubated with Alexa Fluor-488-labeled secondary antibodies for 1 h at room temperature. Slides were washed in PBS again and mounted in the ProLong Diamond mounting media (Invitrogen).

For immunocytochemistry, SRA cells were cultured on glass-bottom cell culture plates (IWAKI Glass, Japan) and fixed with 4% paraformaldehyde in PBS for 10 min, followed by washing with PBS. Fixed cells were permeabilized with PBS containing 0.2% Triton X-100 for 5 min. After blocking with 1 mg/mL BSA in PBST/0.1% Tween-20, cells were incubated with anti-αSMA (ab32575, 1:500; abcam) or anti-fibronectin (F6140, 1:100; clone:FN-3E2, Sigma-Aldrich) in PBS/0.1% Tween-20 for overnight. After washing with PBS, cells were then stained with fluorescent-labeled secondary antibodies. The nuclei were counterstained with NucBlue DAPI stain (Invitrogen). Cells were washed in PBS and then mounted in ProLong Diamond (Invitrogen). Confocal images of fluorescein-stained samples were captured using an LSM 710 confocal microscope (Carl Zeiss).

### Statistical and image analyses

Data are expressed as mean±standard errors of the mean. Statistical analyses were performed using the R package (version 3.4.3; http://www.r-project.org). Pairwise differences between homoscedastic samples were identified using Student's *t*-test, and those between heteroscedastic samples were identified using Welch's *t*-test. Image analysis and quantification of cell fluorescence intensities in immunostaining experiments were performed using the Fiji package of ImageJ (http://fiji.sc/).

## Supplementary Material

Supplementary information
