# Supplementary material for: Ultraviolet B-induced Otx2 expression in lens epithelial cells promotes epithelial–mesenchymal transition
Source: Biol Open. 2019 Feb 4;8(2):bio035691. doi: 10.1242/bio.035691 (PMC6398467; doi:10.1242/bio.035691)
Supplement: Supplementary information [file biolopen-8-035691-s1.pdf]

**Table S1. Primers used in this study**

| purpose | Name | sequence |
|---|---|---|
| RT-qPCR | mouse Otx2 Forward | 5’-GGCCGACTTTGCGCCTCCAA-3’ |
| | mouse Otx2 Reverse | 5’-TCCCTTCGCTGTTTCCGGGG-3’ |
| | human OTX2 Forward | 5’-GAAGAGCTAAGTGCCGCCAA-3’ |
| | human OTX2 Reverse | 5’-TCGGGACTGAGGTGCTAGAG-3’ |
| | human αSMA Forward | 5’-CTATGCCTCTGGACGCACAACT-3’ |
| | human αSMA Reverse | 5’-CAGATCCAGACGCATGATGGCA-3’ |
| | human γSMA Forward | 5’-CTGCCATGTACGTCGCCATTCA-3’ |
| | human γSMA Reverse | 5’-GACATTGTGGGTGACGCCATCA-3’ |
| | human PROX1 Forward | 5’-CTGAAGACCTACTTCTCCGACG-3’ |
| | human PROX1 Reverse | 5’-GATGGCTTGACGTGCGTACTTC-3’ |
| | human CRYAA Forward | 5’-CAGACCGTCTTCCTCCAACC-3’ |
| | human CRYAA Reverse | 5’-GGGTTGCTTGTATCACCTGC-3’ |
| | human DNASE2B Forward | 5’-GCCAGCTCATCAGAGATTCCTG-3’ |
| | human DNASE2B Reverse | 5’-TGAGCCATCCAGGCTGCAAAGA-3’ |
| vector construction | human Otx2 Forward | 5’-gGAATTCGCCACCatgatgtcttatcttaagcaaccg-3’ |
| | human Otx2 Reverse | 5’-cgGGATCCtcacaaaacctggaatttccacga-3’ |

Biology Open • Supplementary information

**Table S2**

**human anterior capsular lens epithelium sample information.** The human lens capsule explants were obtained from cataract surgery performed at the Ophthalmology, Kanazawa Medical University Hospital, Kanazawa, with approval from the Kanazawa Medical University Research Ethics Committee (License No. G134). Samples were subjected to RT-qPCR analysis with or without UVB-irradiation as described in Materials and Methods section. Sample information of each participant's age, gender, sample obtained from right or left eyes, and Otx2 expression were indicated. As indicated in Supplemented table 2, 4 of the 6 samples showed increased-Otx2 expression by UVB-irradiation, whereas 1 sample showed no-change and 1 sample showed decreased expression. Interestingly, endogenous Otx2 expression in these 2 nonresponsive samples were much higher than UVB-responsive samples (data not shown).

| Donor# | Age | Gender | Right/Left | Otx2 expression after UVB-irradiation |
|---|---|---|---|---|
| 1 | 71 | F | R | increased |
| 2 | 70 | M | R | decreased |
| 3 | 70 | F | L | no-change |
| 4 | 86 | F | L | increased |
| 5 | 51 | M | L | increased |
| 6 | 47 | F | R | increased |

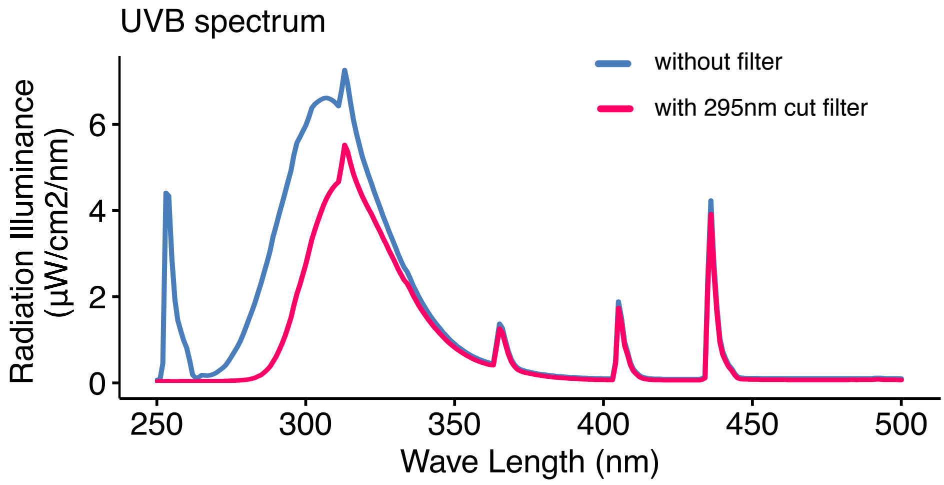


**Fig. S1. UV wave spectrum with 295nm band cut filter.** The UVB wave spectrum of the light source used in the Fig. 2d were analyzed. UVB light spectrum with 295 nm band cut filter (red line), and without filter (blue line) were shown.

Biology Open • Supplementary information

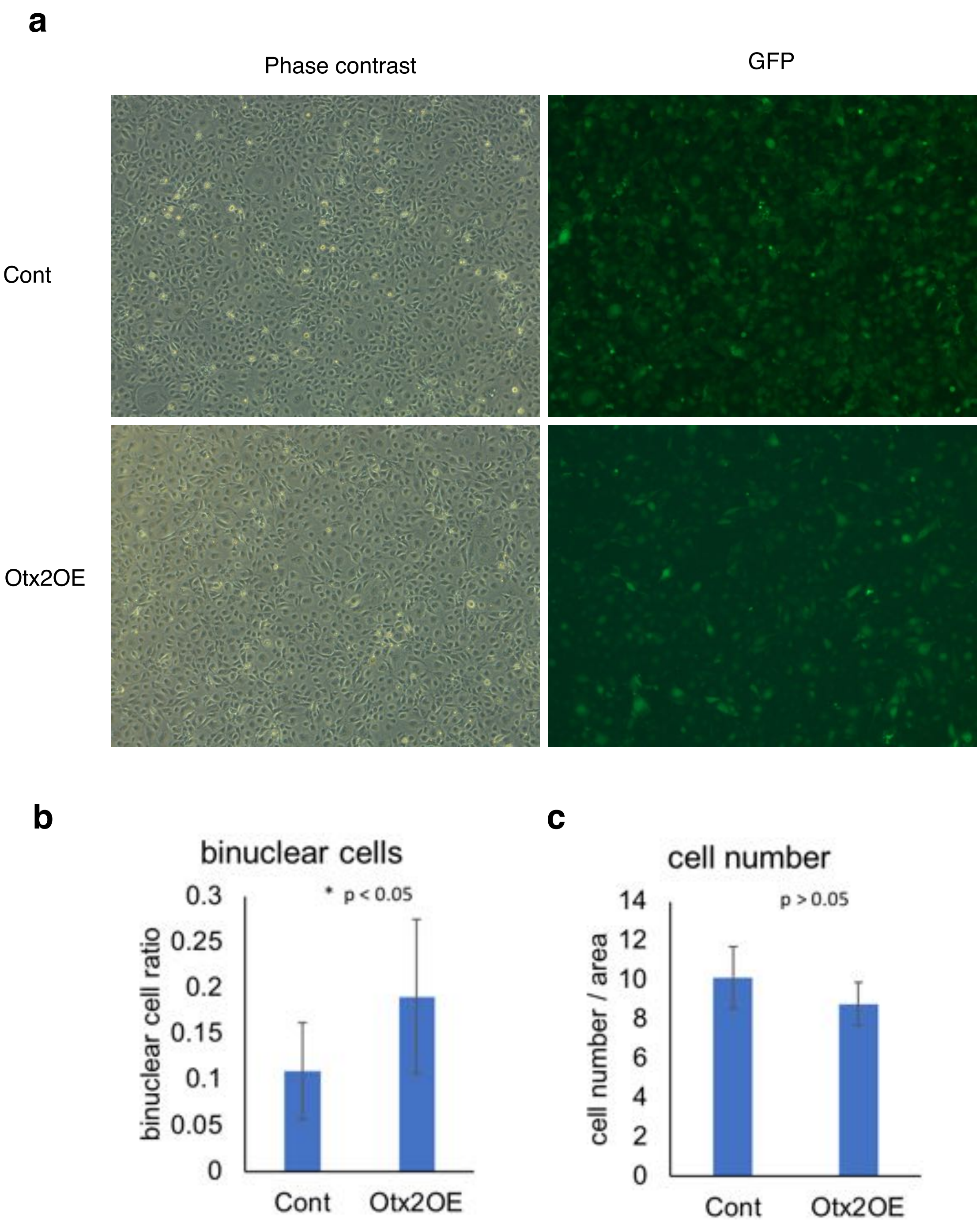


**Fig. S2. Lentiviral transduction efficiencies and cell number.** SRA cells were lentiviraly transduced with control vector (Cont) or Otx2 expression vector (Otx2OE). Two days after transduction, cells were detached and inoculated on glass bottom chamber for fluorescent imaging, and phase contrast images and GFP-fluorescent images were captured (a). After fixation and stained with DAPI, the number of binuclear cells and total cell number were counted and binuclear cell ratio (b) and total cell number (c) were quantified (b).

Biology Open • Supplementary information
